# Cervical metastasis of gingival carcinoma misdiagnosed as branchiogenic carcinoma, a rare entity - report of a case and review of literature

**DOI:** 10.1186/s12903-017-0435-9

**Published:** 2017-11-28

**Authors:** Qingjia Sun, Mingxing Chen, Yuxin Sun, Xi Chen, Hongjun Xu, Lingjun Rong, Qiong Wu, Dongdong Zhu

**Affiliations:** Department of Otorhinolaryngology Head and Neck Surgery, The China-Japan Union Hospital of Jilin University, Xiantai Street 126, Changchun, 130033 China

**Keywords:** Gingival cervical cystic metastatic carcinoma, Branchiogenic carcinoma, Cystic metastasis from occult primary lesions, Nasopharyngeal carcinoma

## Abstract

**Background:**

A cervical cystic mass is associated with a number of pathologies that present with similar symptoms. These conditions are difficult to differentiate using fine-needle aspiration (FNA), ultrasound (US), computed tomography (CT) and magnetic resonance imaging (MRI). Another dilemma in the differential diagnosis of cervical cystic masses is due to the controversies associated with the existence of branchiogenic carcinoma (BC). BC is an extremely rare disease that must be differentiated from other conditions presenting with cervical cystic masses, especially cystic metastasis from occult primary lesions.

**Case presentation:**

We present a case report of a right cervical cystic metastasis from a significantly small squamous cell carcinoma primary gingival lesion misdiagnosed as BC by histopathology. A 62-year-old female presented with a painless progressively enlarging cervical mass at the anterior edge of the sternocleidomastoid muscle in the right submandibular region. Preoperative MRI and US revealed a well-defined cystic round mass. Postoperative histological examination indicated BC. Positron emission tomography/computed tomography (PET/CT) revealed high 18F–FDG (18F 2-fluoro-2-deoxy-D-glucose) uptake in surgical regions with a SUV (standard uptake value) max 4.0 and ipsilateral nasopharynx with a SUVmax 4.4, without any distant metastasis. Pathologic results revealed nasopharyngeal lymphadenosis. Considering the low incidence of BC and the limitation of diagnosis in one institution, the patient was referred to another hospital. Physical examination detected a significantly small neoplasm (~3 mm diameter) in the right lower gingiva. Histopathological examination of the neoplasm revealed a well-differentiated squamous cell carcinoma. Surgery, including a partial mandibulectomy and modified neck dissection (neck level I–V and submental lymph nodes) were undertaken. Postoperative histopathological results revealed a well-differentiated squamous cell carcinoma of right lower gingiva and two metastatic lymph nodes in the 18 lymph nodes of level II. A month later, recurrence occurred in the right cervical level II. The patient was placed on postoperative concurrent chemo-radiotherapy and supportive care. The patient suffered from cachexia and survived for only six months after surgery.

**Conclusions:**

In cases of cervical cystic masses that appear after the age of 40, clinicians should bear in mind that occult primary lesions should be excluded and examination of the gingiva should be undertaken. PET/CT has a limited role in identifying small occult primary lesions and a comprehensive physical examination must be carefully performed.

## Background

The majority of head and neck carcinomas are squamous cell carcinomas with a high incidence of recurrence and metastasis [1, 2]. Survival is reduced if metastasis has occurred [3, 4]. Cervical lymph node metastases of head and neck carcinomas have been extensively studied, with the majority developing as solid masses [1, 5]. However, the preoperative differential diagnosis of cervical cystic metastasis is challenging, especially in carcinoma of unknown primary (CUP), which account for 3–9% of all head and neck carcinomas [6]. Cervical cystic masses are associated with a number of conditions that often present similar symptoms, including branchial cleft cysts (BCC), branchiogenic carcinomas (BC) and cervical cystic CUP [7–9]. However, these conditions are difficult to differentiate from each other by fine-needle aspiration (FNA), ultrasound (US), computed tomography (CT) and magnetic resonance imaging (MRI) [8]. Among these lesions, BCC is the most common, which results from the incomplete obliteration of the embryonal branchial apparatus and is lined by stratified squamous epithelium histologically [10, 11]. Although BC are associated with cystic masses, there is much controversy surrounding such a diagnosis [12, 13]. BC is an extremely rare disease, which was initially believed to be a malignant transformation of the epithelium within the branchial cyst walls [10]. In 1950, Martin et al. [14] proposed strict diagnostic criteria for BC, which were later modified by Khafif et al. in 1989 [15]. At present, however, diagnosis of BC remains a topical debate. The lack of multi-center analysis and inadequate number of samples are contributory factors that impede precise diagnosis.

The key method for BC diagnosis is dependent on surgical excisional biopsy. Although FNA has a high accuracy (>90%) for the diagnosis of cervical solid masses, false-negative rates for cystic masses are as high as 38–63% [16]. Small cancerous lesions may remain unidentified using 18F 2-fluoro-2-deoxy-D-glucose (18 F-FDG) positron emission tomography/computed tomography (PET/CT) [17] and hence limit its role in the differentiation between cervical cystic CUP and BC. Therefore, cervical cystic CUP may easily be misdiagnosed as BC [8] or BCC, preoperatively [18].

In the present study, we report a case of right cervical cystic metastasis from a significantly small squamous cell carcinoma primary gingival lesion, misdiagnosed as BC by histopathology and nasopharyngeal carcinoma by PET/CT. The misdiagnosis impacted on the prognosis and quality of life of the patient. Squamous cell carcinoma of the gingiva is less common than carcinoma of other tissues in the oral cavity; however, there are no differences in initial symptoms [19]. The management of oral squamous cell carcinoma is not straightforward and may involve surgery, radiotherapy, or a combination of both [19, 20]. However, recommended therapeutic approaches for the management of cervical CUP are surgical neck dissection, radiotherapy or neck dissection and comprehensive radiotherapy in combination [6]. By contrast, treatment of BC has been undertaken by both excising a single mass alone [21], or by complete surgical excision followed by adjunct radiotherapy [22–24]. The management of the malignancy therefore depends on distinct guidelines. Therefore, accurate preoperative diagnosis is essential for treatment planning and improving outcomes and prognoses.

## Case presentation

**Fig. 1:**
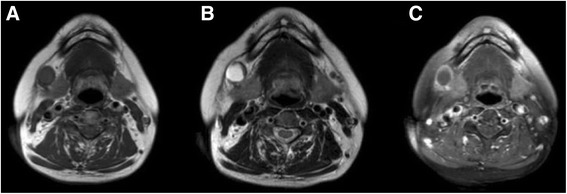
Preoperative MRI of the neck: a well-defined round mass proximal to the anterolateral side of the right submandibular gland, **a** T_1_WI revealed a well-distributed intermediate signal; **b** T_2_WI revealed a high signal; and **c** contrast-enhanced T_1_WI revealed an obvious enhancement of the peripheral wall

A 62-year-old female was referred to the Department of Otorhinolaryngology Head and Neck Surgery of the China–Japan Union Hospital of Jilin University, China, in March 2016. The patient presented with a two-month history of a painless and progressively enlarged unilateral mass on the right side of the neck. Clinical examination revealed a submandibular swelling located at the anterior edge of the sternocleidomastoid muscle on the right side of neck. The female had no history of smoking or alcohol consumption. Endoscopic examination, including laryngoscopy, rhinoscopy, and endotoscopy, did not detect any pathology in the nasal cavity, nasopharynx, oropharynx, hypopharynx or larynx. Ultrasonic examination revealed a cystic hypoechoic change, approximately 1.5 cm, in the right submandibular region. MRI of neck revealed a well-defined round mass (~1.5 × 1.7 × 1.6 cm) proximal to the anterolateral side of the right submandibular gland. The mass had a well-distributed intermediate signal at T_1_WI and a high signal at T_2_WI, with an obvious enhancement of the peripheral wall in contrast-enhanced T_1_WI (Fig. 1). These images strongly suggested a cervical cystic lesion with the characteristics of a benign mass.

Based on the detailed history, clinical examination and investigations, surgical excision of the lesion was planned. The provisional diagnosis of BCC and treatment options were explained to the patient and informed written consent was obtained for the proposed treatment. The case study and treatment plan was approved by the institutional review board and ethics committee of The China-Japan Union Hospital of Jilin University.

**Fig. 2:**
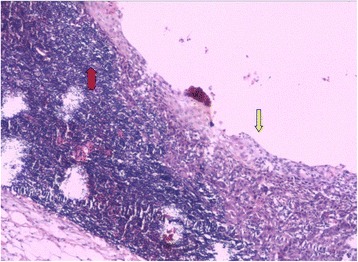
The histopathologic examination showed that the resected mass of lateral neck appeared as a cystic structure overlapped by lymphoid tissue (red arrow) with an internal surface lined by squamous cell carcinoma epithelium (yellow arrow) (HE ×40)

**Fig. 3:**
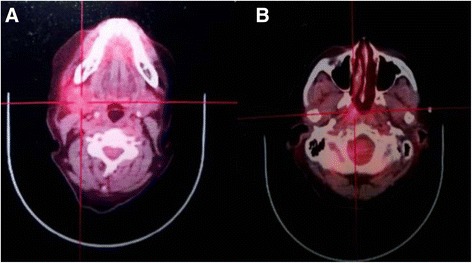
PET/CT revealed high FDG uptake in **a** surgical regions and **b** ipsilateral nasopharynx

The mass was excised completely, using a right-neck lateral approach under general anesthesia. During surgery, the lesion was highly pigmented and appeared as a well-defined ovoid cystic mass with little adhesion to marginal mandibular branch of the facial nerve. Frozen section analysis indicated a diagnosis of squamous cell carcinoma with a significant possibility of BC rather than a metastatic lymph node. We, therefore, chose conservative resection of the lesion. Postoperative histopathologic examination was performed by two senior pathologists independently under double-blind conditions. The resected mass appeared to be cystic, overlapped by lymphoid tissues. The internal surface was lined by thin-layered squamous epithelium with severe dysplasia and had characteristic features of BC (Fig. 2). Considering the low incidence of BC, PET/CT was performed to differentiate cervical metastasis of unknown primary lesion. The results of the scan showed high FDG uptake in surgical regions with SUVmax 4.0 and homolateral nasopharynx with SUVmax 4.4, without any evidence of distant metastasis (Fig. 3), which suggested nasopharyngeal malignancy. For diagnosis of nasopharyngeal carcinoma, the patient agreed to undergo nasopharyngeal biopsy twice by two senior otolaryngologists. All histopathologic examinations revealed lymphadenosis. Considering the limited resources of the primary institution, the patient was referred to a specialist hospital, The Cancer Institute & Hospital, Chinese Academy of Medical Sciences. In an outpatient clinic, physical examination was performed and showed a significantly small neoplasm (~3 mm at its largest diameter) in the right lower gingiva. Initial pathological diagnosis of the small neoplasm was conducted using a forceps biopsy technique and revealed a well-differentiated squamous cell carcinoma. Following detailed study of the case and investigations, a final diagnosis of gingival carcinoma with neck metastasis was made. Surgery including partial resection of the mandible and modified neck resection (neck level I–V and submental lymph nodes) was performed. Postoperative pathology examination revealed a well-differentiated squamous cell carcinoma of the right lower gingiva and two metastatic lymph nodes of the 18 lymph nodes of level II. A month later, recurrence occurred in the right cervical level II and the patient underwent postoperative concurrent chemo-radiotherapy. The patient became cachectic and lived for only six months.

## Discussion

In the present case, we shared the experience of the misdiagnosis between cervical metastasis of gingival carcinoma and BC. One possible reason for misdiagnosis may be due to our lack of experience of cervical cystic disease. Cervical cystic metastasis remains a challenging issue for preoperative diagnosis, with an incidence ranging from 33% to 62% [10]. Cervical cystic diseases may be associated with BCC, BC, cervical cystic CUP, cystic necrotic schwannoma, lymph node with necrotic granulomatous inflammation, lymphangioma, venous malformation, lymphoma with cystic degeneration, metastatic papillary thyroid carcinoma [10, 25], or malignant ectopic thyroid glands [26, 27]. The diagnosis of BC remains controversial and excluding other potential similar presentations, such as cervical cystic CUP, is paramount. In 1950, Martin et al. [14] proposed strict diagnostic criteria for BC as follows:

1. Cervical tumors must be located along the anterior border of the sternocleidomastoid muscle from the tragus to the clavicle.
2. The histological appearance of the growth must be consistent with an origin in the tissues present in the branchial vestiga.
3. The patient must be followed up for at least five years without appearance of any occult primary tumors.
4. Histological evidence of the developing cancer must be found in the wall of an epithelial cyst situated in the lateral aspect of the neck.

However, many authors argue that it is difficult to meet the third criterion, because the duration of diagnosis is too long for therapy or patients who underwent postoperative radiotherapy, which may affect appearance of occult primary [28]. In 1989, Khafif et al. [26] modified these criteria and attenuated the role of follow up and emphasized the absence of an identifiable primary as well as clear histological identification of the nature of the tumor. To date, the incidence of BC is extremely rare [16].

The second reason for misdiagnosis may be due to the lack of comprehensive physical examinations and over dependence on radiological examinations, such as PET/CT. The present case, presenting with BC histologically, was finally diagnosed as cervical cystic metastasis with occult primary originating from gingiva. A similar case has been reported by Zhang et al. [29], where a case of upper cervical cystic mass appearing as BC histologically was found. It had metastasized from the gingiva and was diagnosed through PET/CT. To the best of our knowledge, the presenting case is the second report of cervical cystic metastasis from occult gingiva primary lesion. In contrast, the approximately 3 mm primary lesion of the present case is too small to detect through PET/CT, compared to the 1 cm lesion in the Zhang et al. report [29]. The role of PET/CT for the detection of CUP has been well described [30]. However, Ferris et al. [17] concluded that PET/CT has a limited role in smaller primary lesions, and the findings could be misleading. Consistent with Ferris et al. [17], the right nasopharynx of the case exhibited high uptake of FDG and the result of the biopsy showed chronic inflammation, as inflammatory lesions can have a high SUV value [31]. In our report, the final detection of occult primary lesions was dependent on oral physical examination. Therefore, careful physical examination should not be ignored in the search for occult primary lesions.

Squamous cell carcinoma of the gingiva is an extremely rare disease with a female predominance [32]. In comparison with other oral squamous cell carcinoma, the cause of gingiva squamous cell carcinoma is rarely associated with smoking, ultraviolet radiation, oncogenic viruses and infection [32]. The treatment of the disease includes surgery and/or radiotherapy, depending on the tumor stage [33]. Gingival squamous cell carcinoma has a variable clinical presentation that allows the disease to be easily misdiagnosed as a benign tumor or other inflammatory responses [34]. It has been reported that prognosis of gingival squamous cell carcinoma is dependent on volume [35]; therefore, early detection is crucial for improving prognosis. It has been reported that 72–90% of cervical squamous cell carcinoma metastases are from Waldeyer’s ring (base of tongue, palatine tonsils and nasopharynx) [18, 36, 37], and other sites, including the larynx, hard palatine, and thyroid gland, are rare [10]. Zhang et al. [8] reported two cases with cervical cystic masses, which were diagnosed as malignant after surgical excision. The final diagnosis was nasopharyngeal carcinoma with neck metastasis determined by biopsy. Papillary carcinoma of ectopic thyroid glands arising from BCC could appear as solitary cervical cystic mass [26, 38]. It has been suggested that cervical cysts in patients older than 40 years of age should raise the possibility of metastases [18], which is consistent with the current case. The present case and Zhang et al.’s report [29] suggests that the oral cavity including the gingiva must be checked in patients suffering from suspected cervical cystic malignancy.

## Conclusions

The incidence of BC is extremely rare. In cases of cervical cystic masses that appear clinically after the age of 40, clinicians should bear in mind that occult primary lesions should be excluded and the examination of the gingiva should not be ignored. PET/CT has a limited role in the identification of small occult primary lesions. Hence, comprehensive physical examinations must be carefully performed to explore lesions at the earliest stage.

## Abbreviations

18F-FDG: 18F 2-fluoro-2-deoxy-D-glucose
BC: Branchiogenic carcinoma
BCC: Branchial cleft cyst
CT: Computed tomography
CUP: Carcinoma of unknown primary
FNA: Fine-needle aspiration
MRI: Magnetic resonance imaging
PET/CT: Positron emission tomography/computed tomography
US: Ultrasound
